# Ultrasmall gold nanoparticles in cancer diagnosis and therapy

**DOI:** 10.7150/thno.42471

**Published:** 2020-03-31

**Authors:** Miao Fan, Yu Han, Shutao Gao, Hongyu Yan, Lingzhi Cao, Zhenhua Li, Xing-Jie Liang, Jinchao Zhang

**Affiliations:** 1 College of Chemistry & Environmental Science, Chemical Biology Key Laboratory of Hebei Province, Key Laboratory of Medicinal Chemistry and Molecular Diagnosis of the Ministry of Education, Hebei University, Baoding 071002, P. R. China;; 2 CAS Key Laboratory for Biological Effects of Nanomaterials and Nanosafety, National Center for Nanoscience and Technology, Beijing 100190, P. R. China.

**Keywords:** ultrasmall gold nanoparticles, theranostics, size, imaging, cancer therapy

## Abstract

Due to their lower systemic toxicity, faster kidney clearance and higher tumor accumulation, ultrasmall gold nanoparticles (less than 10 nm in diameter) have been proved to be promising in biomedical applications. However, their potential applications in cancer imaging and treatment have not been reviewed yet. This review summarizes the efforts to develop systems based on ultrasmall gold nanoparticles for use in cancer diagnosis and therapy. First, we describe the methods for controlling the size and surface functionalization of ultrasmall gold nanoparticles. Second, we review the research on ultrasmall gold nanoparticles in cancer imaging and treatment. Specifically, we focus on the applications of ultrasmall gold nanoparticles in tumor visualization and bioimaging in different fields such as magnetic resonance imaging, photoacoustic imaging, fluorescence imaging, and X-ray scatter imaging. We also highlight the applications of ultrasmall gold nanoparticles in tumor chemotherapy, radiotherapy, photodynamic therapy and gene therapy.

## Introduction

Over the last few decades, the use of gold nanoparticles (GNs) in biomedical applications has piqued interest owing to their intrinsic properties, which make them suitable for the diagnosis and treatment of cancer. GNs, like many precious metals, have a unique optical property known as surface plasmon resonance (SPR), which allows them to be used in near-infrared (NIR)-resonant biomedical imaging modalities such as magnetic resonance imaging (MRI), photoacoustic imaging (PAI), fluorescence imaging and X-ray scatter imaging 1, 2. GNs also generate heat when exposed to NIR laser light, which makes them suitable for the photothermal treatment of cancer 3, 4. In addition, GNs have low toxicity and are nonimmunogenic by nature. Their synthesis methods are simple, and their size, shape and surface modifications can be readily controlled. All these properties mean that GNs can be functionalized in many different ways for local hyperthermia of cancer tissue and delivery of multiple drugs in a controlled and targeted manner 5. Based on these excellent characteristics, gold nanostructures have been studied and administered in phase I and II clinical trials for cancer treatment 6. Size is one of the key parameters of GNs that influence half-life time, systemic toxicity, tumor accumulation and so on, which are important properties for imaging and therapeutic applications. As the range of applications of GNs continues to increase, it is necessary to better understand the biological effects of GNs of different sizes.

The promising application of GNs in cancer treatment depends largely on their ability to penetrate tumor tissues. The studies of Liang et al. and Hong et al. showed that ultrasmall GNs displayed obvious advantages in penetration of tumor tissue in mice 7, 8. Liang et al. quantified the cellular uptake of 2, 4, and 6 nm core GNs featuring neutral (zwitterionic), anionic, and cationic headgroups. They found that changes in particle size and surface ligand coverage can be used synergistically to control cell uptake 9. Lei et al. found that GNs inhibit the CpG oligodeoxynucleotide (CpG-ODN)-induced production of TNF-α in a manner that depended on the concentration and size of GNs. Specifically, GNs (4 nm) are more potent than larger GNs (11, 19, 35 or 45 nm) 10. These studies confirmed that the size of GNs affects their cellular uptake and that smaller GNs are taken up more readily. On this basis, researchers also analyzed the ability of GNs to enter the nucleus. Kumar et al. and Huang et al. proved that GNs (2 nm and 6 nm) can effectively penetrate into the nucleus 7, 11. In addition, smaller GNs show lower toxicity than large GNs. Some research reports indicated that GNs can eventually be removed from the body through the glomerular filtration system, instead of accumulating extensively in various cells, tissues and organs 12-14. Zheng et al. confirmed that the particle size can influence the renal clearance efficiency, and GNs (6 nm) could be cleared from the blood by filtration through the kidney to the bladder 15. By designing a gold nanocluster disease detection platform, Stevens et al. indirectly demonstrated that ultrasmall gold nanoparticles could be completely cleared from the body through the liver and kidney 16. Therefore, the intrinsic properties of ultrasmall GNs provide more potential for their use in cancer diagnosis and therapy.

In this review, we focus specifically on ultrasmall GNs, which have diameters of less than 10 nm. We will firstly discuss the methods for synthesizing and surface functionalization of ultrasmall GNs. The use of ultrasmall GNs in imaging techniques for cancer diagnosis will be reviewed briefly. The applications of ultrasmall GNs in cancer therapy, including chemotherapy, gene therapy, radiotherapy and so on, will be discussed in more detail. This information may be significant in the further development of clinical applications for GNs.

## Synthesis and surface functionalization

The wet synthesis method, which is commonly used for preparing larger GNs, has also been used to prepare ultrasmall GNs 17, 18. Here we will focus more on methods that effectively control the size of ultrasmall GNs. The simplest and most commonly used method for generating different sizes of ultrasmall GNs is to change the ratio of reactants. Jaffray et al. prepared different sizes of GNs by changing the ratio between the gold salt and the reducing agent 19. Liang et al. fabricated different sizes of GNs by changing the volume of sodium citrate solution 20, while Lei et al. changed the amount of trisodium citrate solution added 10. Huang et al. generated GNs with different diameters ranging from 3 to 100 nm by changing the dosage of seed colloid added 21. In addition, there are two special methods that can be used to regulate the size of GNs. One way is to control the size of the gold core by introducing ligands. Schmid et al. firstly proposed this method in 1981 to formulate Au_55_(PPh_3_)_12_Cl_6_, which has been widely investigated in models of metal-based catalysis 22. Phosphine-stabilized GNs have unparalleled small size and narrow dispersity (1.4 nm) 23. On this basis, Hutchison et al. adjusted the diameter of the gold core from 1.4 nm to 10 nm through ligand exchange reaction 24. Murray et al. finely adjusted the size of ultrasmall GNs between 1.5 nm and 5.2 nm by varying the Au: dodecanethiolate ratio and the temperature and rate of the reduction 25. The second method for controlling the size of GNs is to utilize a unique carrier as the dispersed inner cavity. Zhang et al. reported a method to control size through the formation of GNs and carbon nanospheres at the same time after a reduction reaction 26. Lu et al. synthesized ultrasmall GNs by *in situ* growth in a two-dimensional mixed ligand metal-organic framework nanosheet 27. These preparation methods provide the basis for biomedical applications of ultrasmall GNs.

The surface modification of ultrasmall GNs is necessary to reduce their surfactant-induced toxicity and increase their biocompatibility. The most important method for functionalization of ultrasmall GNs is the thiol gold reaction, which depends on the strong affinity between thiols and Au. GNs with hexadecyl trimethyl ammonium bromide (CTAB) as surfactant can be detoxified and stably dispersed by displacing the CTAB with thiolated species 1, 28, 29. Liang et al. obtained stably dispersed ultrasmall GNs (2, 4, and 6 nm) coated with zwitterionic ligands via gold-sulfur bonding 9, while Garcia et al. obtained ultrasmall GNs (5 nm) that were stabilized with double-pyridine salts for cancer therapy 30. Wu et al. modified ultrasmall GNs (≈12 nm) with a conjugate of folic acid (FA) and reduced bovine serum albumin for photothermal therapy and dual-modal imaging 31. Some other methods for surface functionalization of ultrasmall GNs (such as electrostatic adsorption) are also based on the thiol gold reaction. Rotello et al. developed a DNA delivery system that combines trimethylammonium mixed monolayer protective cluster modified GNs (2 nm) and DNA through electrostatic interaction 32. Rotello et al. also synthesized particles with gold cores (2 nm) and triethylenetetramine-terminated dendron ligands, which further interacted electrostatically with negatively charged siRNA 33. The universality of the gold sulfur bond provides the basis for the surface functionalization of ultrasmall GNs, which opens up more possibilities for the use of ultrasmall GNs as a versatile cancer theranostic platform.

## Ultrasmall gold nanoparticles in cancer diagnosis

### Magnetic resonance imaging

**Figure 1:**
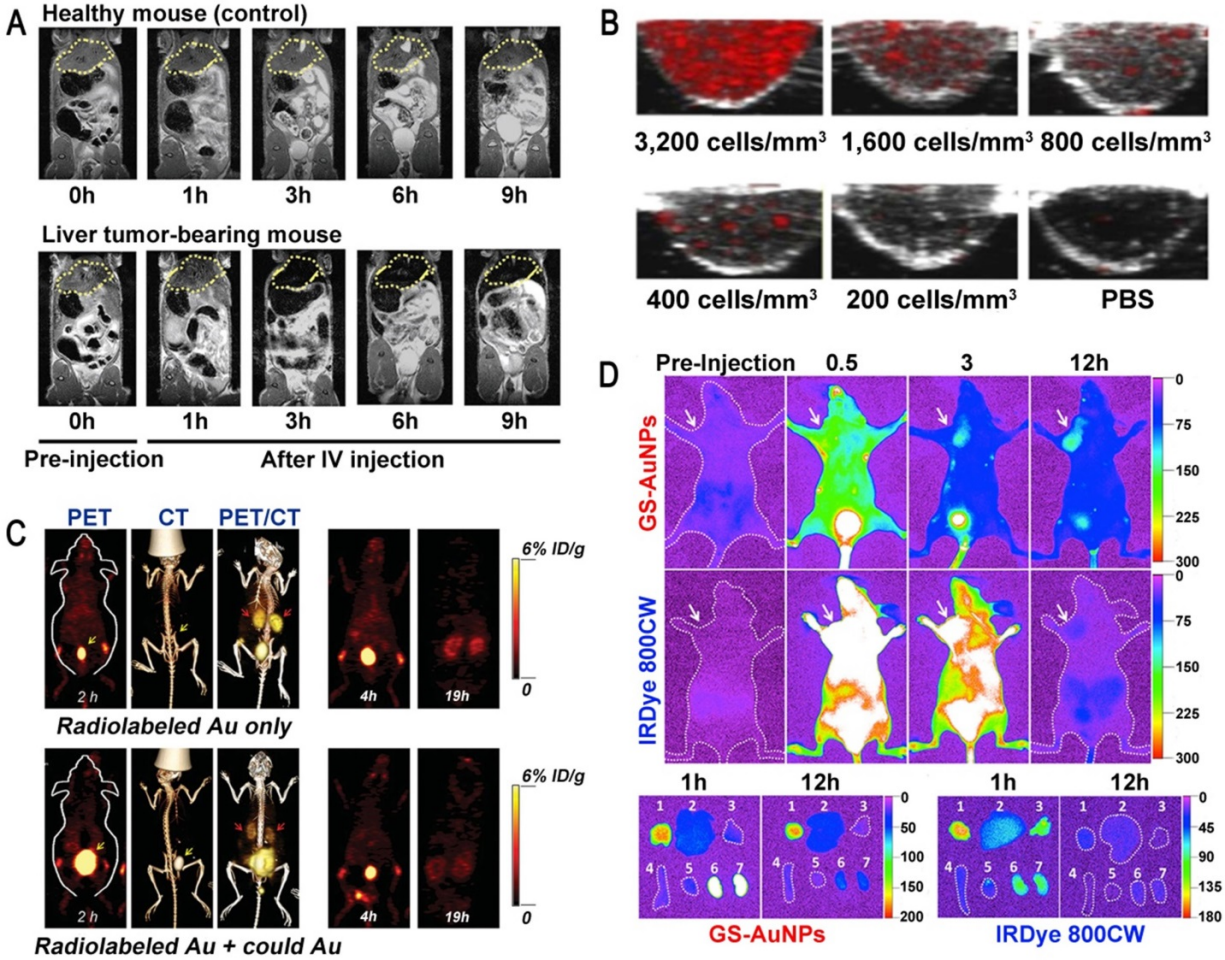
The use of ultrasmall GNs in cancer diagnosis. (A) T2-weighted MR images of a healthy mouse (top) and a mouse bearing a liver tumor (MDA-MB-468; bottom) using superparamagnetic gold-nanoparticle clusters as a contrast agent. Adapted with permission from 12, copyright 2017 WILEY-VCH Verlag GmbH & Co. KGaA, Weinheim. (B) Representative PAI cross-sectional images of A431 cells labeled with different concentrations of 5 nm ultrasmall GNs for 10 hours. Adapted with permission from 42, copyright 2019 Optical Society of America. (C) PET, CT, and PET/CT imaging of BALB/c mice injected with only radiolabeled GSH-GNs or radiolabeled GSH-GNs and cold Au. Adapted with permission from 46, copyright 2016 Wiley-VCH Verlag GmbH & Co. KGaA, Weinheim. (D) Representative *in vivo* and *ex vivo* NIR fluorescence images of MCF-7 tumor-bearing mice *i.v.*-injected with GSH-GNs and IRDye 800CW. Adapted with permission from 49, copyright 2013 American Chemical Society.

Magnetic resonance imaging (MRI) is an examination technique based on the principle of nuclear spin. MRI is of great value in the diagnosis of degenerative diseases. Superparamagnetic inorganic nanoparticles can be used in MRI and magnetic hyperthermia therapy, but their potential toxicity to the human body limits their clinical application 34, 35. For example, the toxicity of reactive oxygen species (ROS) induced by superparamagnetic iron oxide nanoparticles (SPIONs) can lead to severe DNA and protein damage as well as inflammation, so the use of SPIONs in MRI is no longer allowed in the clinic 36, 37. Ultrasmall GNs have no such toxicity and have been widely used for *in vivo* medical applications. Lee et al. synthesized ultrasmall GNs with a particle size of 1.4 nm using the hepatitis B virus (HBV) core protein as a scaffold. These GNs had significant MRI and magnetic hyperthermia therapy effects on both subcutaneous and deep-tissue tumors in live mice 12. Importantly, the use of ultrasmall GNs could circumvent the tissue damage caused by long-term retention of inorganic nanoparticles in the kidney (Figure 1A). This indicates that ultrasmall GNs may have broad applications in the clinical diagnosis of cancer.

### Photoacoustic imaging

Photoacoustic imaging (PAI) is a new non-invasive and non-ionizing biomedical imaging method. In PAI, biological tissues are irradiated with non-ionizing laser pulses, and the endogenous molecules (such as hemoglobin or melanin) or exogenous contrast agents absorb light energy. This optical absorption, which is associated with the physiological properties of the tissue, is converted into ultrasonic signals carrying the optical absorption characteristics of the tissue. 2D or 3D optical absorption distribution images can be reconstructed by instrument detection and data analysis 38. GNs have a high absorption cross-section, which is thousands of times higher than that of common organic dyes such as rhodamine-6G or indocyanine green 39. Because of this excellent performance, GNs have been successfully used as molecularly targeted contrast agents for PAI in animal models. However, GNs with larger diameter are not efficiently excreted through the kidney, which limits their application in the clinic 40, 41. Therefore, it is necessary to develop specific ultrasmall GNs for PAI. Sokolov et al. coupled a monoclonal anti-EGFR (epidermal growth factor receptor; EGFR) antibody to GNs with particle sizes of about 5 nm and 40 nm, and used them to label cancer cells 42. The results showed that the 5 nm GNs had strong near-infrared absorption and maintained the same PA signal as the 40 nm GNs. Surprisingly, the 5 nm GNs showed outstanding tissue penetration and *in vivo* clearance ability due to their ultrasmall diameter (Figure 1B). These results suggested that ultrasmall GNs can provide a viable option for sensitive PAI of cancer cells *in vivo*.

### Positron emission tomography imaging

Positron emission tomography (PET) is a relatively advanced clinical imaging technique based on positrons released after decay of positron radionuclides, which is especially suitable for early diagnosis of diseases. At present, PET is an important technology for diagnosing tumors. Although a large number of nanoparticles have been reported for the detection and diagnosis of cancer in animal models, their accumulation in tissues means that they cannot be used in clinical applications 43, 44. Ultrasmall GSH-coated luminescent GNs (3.5 nm) can be cleared by the kidney and also have many advantages in other cancer-targeting imaging techniques. They have been used as a contrast agent in single-photon emission computed tomography (SPECT) for depth imaging and quantification of tissue, but their development was limited due to the low temporal resolution of SPECT 45. On this basis, some researchers proposed that ultrasmall GNs can also be applied to the more advanced nuclear magnetic imaging technology PET. Cai et al. utilized ^64^Cu-labeled ultrasmall GNs (2.5 nm) as PET imaging contrast agents to assess kidney function in mice for the potential diagnosis of kidney disease 46. This work demonstrated the rapid clearance of GSH-coated GNs by the kidney and the outstanding performance of PET in accurate, non-invasive acquisition of organ kinetics (Figure 1C). It is expected that the use of modified ultrasmall GNs as a PET imaging contrast agent can also be expanded for the detection and diagnosis of cancer.

### Fluorescence imaging

Fluorescence is a common phenomenon in nature. The principle of fluorescence imaging is based on the linear relationship between the intensity of the fluorescent signal emitted by the excited fluorescent material and the amount of fluorescent substance in a certain range. GNs possess many well-known optical properties and can passively accumulate at tumor sites, which makes them even more promising than small drug molecules in early cancer diagnosis. However, when used in tumor detection, GNs also accumulate extensively in organs of the reticular endothelial system (RES) (liver, spleen, etc.), which lowers their targeting specificity and hinders their clinical application 47. In order to fully exploit the enhanced permeability and retention (EPR) effect of tumors, it is necessary to use ultrasmall GNs, because they can stay at higher concentrations in the plasma for more than 6 hours, and further escape kidney filtration 48. Zheng et al. performed fluorescence imaging in MCF-7 tumor-bearing mice with ultrasmall GNs (2.5 nm) modified by glutathione (GSH), and compared them with the small dye molecule IRDye 800CW 49. The results showed that the ultrasmall GNs, as an effective fluorescent reagent, were more suitable for tumor detection than IRDye 800CW. The GSH-modified ultrasmall GNs enhanced the EPR effect and were further cleared from normal tissues soon after imaging (Figure 1D). This result confirmed the great potential of renal-clearable ultrasmall fluorescent GNs in cancer diagnosis.

### X-ray scatter imaging

In recent years, X-ray scatter imaging has become a relatively popular imaging technology. Similar to X-ray imaging technology, the principle of X-ray scatter imaging is based on the difference in X-ray penetration intensity caused by the difference in tissue density and thickness. It is different from typical absorption X-ray imaging and CT imaging in that there is no need to inject a large amount of contrast agent into the patient 50, 51. When imaging the human body, it is necessary to use a contrast agent such as iodine-based nanoparticles. It is well known that GNs have a higher atomic number and X-ray absorption coefficient than iodine-based nanoparticles 52. These properties, coupled with their low toxicity, makes GNs ideal candidates for use as an X-ray contrast agent 53, 54. With the aim of improving the detection of early hepatocellular carcinoma (HCC), Rose-Petruck et al. synthesized GNs coated with a polyelectrolyte for use as a contrast agent in X-ray scattering imaging of HCC cell pellets. They compared the imaging effect of 10 nm and 50 nm GNs 52. The results showed that the X-ray scatter imaging technique with ultrasmall GNs as the contrast medium had higher sensitivity and was obviously better than the traditional X-ray imaging technique in the detection of small clusters of HCC cells. Smilowitz et al. used ultrasmall GNs to improve X-ray imaging and radiotherapy in mice. They found that more ultrasmall GNs accumulated at the tumor site and higher contrast appeared because of the higher vascular permeability in the tumor 55. These results provide evidence that ultrasmall GNs can be used to improve the tumor diagnosis rate. Thus, GNs are expected to play a more important role in the field of X-ray scattering imaging.

## Ultrasmall gold nanoparticles in cancer therapy

### Chemotherapy

**Figure 2:**
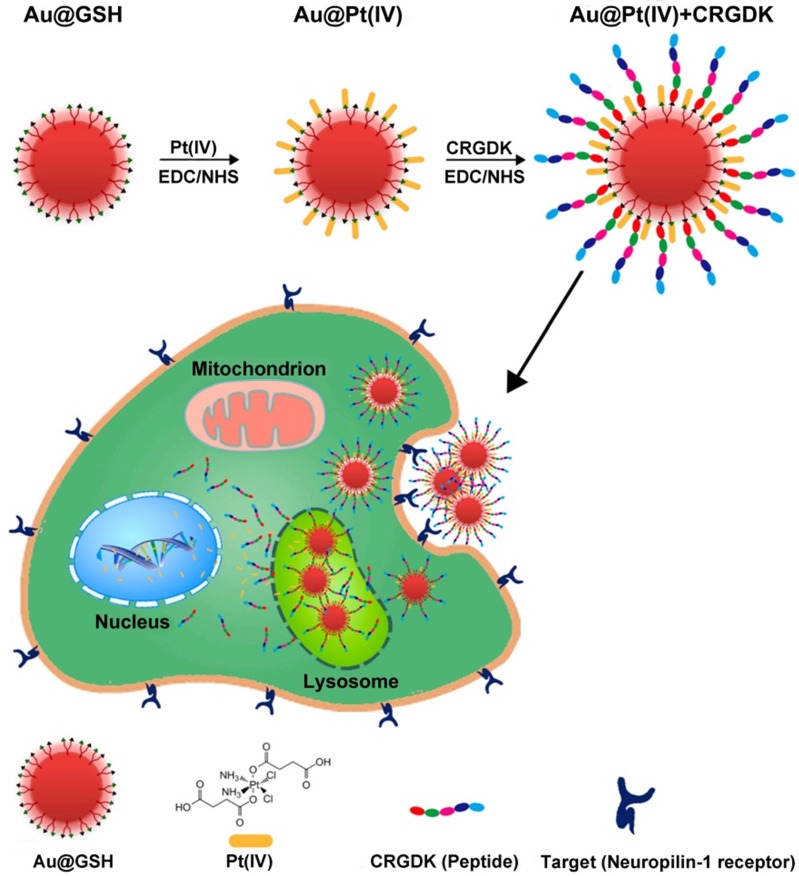
Ultrasmall GNs in cancer chemotherapy. Schematic representation of the synthesis and mechanism of action of GNs targeting the neuropilin-1 receptor. The scheme for the functionalization of GSH-GNs (Au-GSH) with the chemotherapeutic drug Pt(IV) and the targeting peptide CRGDK is shown in the top part. The fully functionalized delivery system is Au@Pt(IV)+CRGDK. The individual components are shown in the bottom part. The middle part shows the interaction between the neuropilin-1 receptor on the prostate cancer cell surface and the targeting ligand on the nanoparticles. This enhances cellular uptake of the nanoparticles by endocytosis and release of active cisplatin into the nucleus. Adapted with permission from 57, copyright 2014 American Chemical Society.

Chemotherapy is the use of chemical drugs to treat cancer. However, chemotherapeutic drugs are inevitably internalized by normal cells and consequently cause toxic side effects. Furthermore, cancer cells can acquire drug resistance by over-expression of drug efflux pumps, increased drug metabolism, and alteration of drug targets. Therefore, a main challenge of chemotherapy is to increase the accumulation of therapeutic drugs in tumor cells, thereby enhancing the efficacy of the treatment and reducing toxic side effects 6. In this respect, it is important to develop efficient drug delivery systems. GN-based nanocarriers are considered to be attractive candidates for delivery of various payloads, as they have low toxicity and high loading capacity 11, 56. Liang et al. developed a unique strategy to deliver a platinum(IV) drug to prostate cancer cells. They constructed glutathione-modified GNs (5.2 nm) carrying the drug and the targeting peptide CRGDK (Cys-Arg-Gly-Asp-Lys), and studied the anticancer effects of this delivery system on prostate cancer cells 57. In this study, the targeting peptide ensured specific binding of the nanoparticles to the neuropilin-1 (Nrp-1) receptor on the surface of the cancer cells, leading to enhanced cellular uptake. Thus, the platinum(IV) drug was specifically delivered to prostate cancer cells, where it exerted its cytotoxic effects. At the same time, the functionalized GNs upregulated the expression of nuclear factor kappa-B (NF-κB) proteins and activated the DNA-binding ability of NF-κB to trigger platinum-induced apoptosis (Figure 2). These GNs, functionalized with platinum(IV) along with a targeting peptide, showed great potential for future anticancer treatment.

**Figure 3:**
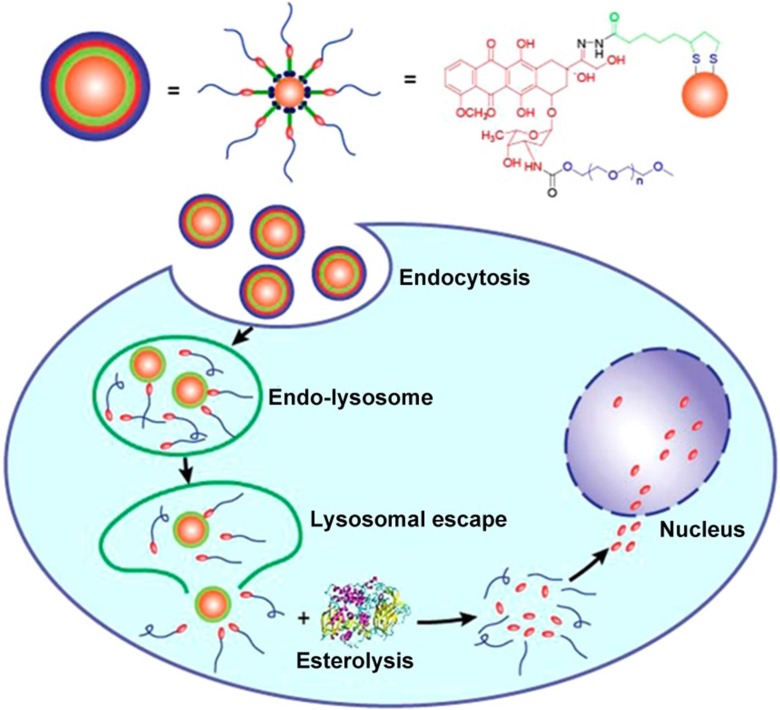
Ultrasmall GNs in cancer chemotherapy. Schematic representation of the synthesis and drug release mechanism of DOX-conjugated GNs. The upper part shows the scheme for preparing DOX-conjugated GNs. The lower part shows the release process after endocytosis of the DOX-conjugated GNs. DOX-mPEG is liberated from the GNs in the acidic lysosomes, and then free DOX is generated in the cytoplasm as a result of esterase activity. Adapted with permission from 61, copyright 2017 American Chemical Society.

Doxorubicin (DOX) is another common chemotherapy drug that effectively induces remission of multiple malignancies. Yook et al. constructed GNs (12 nm) conjugated with DOX and anti-PD-L1 for the targeted chemo- and photothermal therapy of colorectal cancer 58. This study demonstrated that the stable GNs have a high affinity with PD-L1-overexpressing CT-26 cells. Intracellular uptake via binding of the anti-PD-L1 antibody to PD-L1 improved intracellular retention and the therapeutic efficacy of DOX. When the nanoparticles were irradiated by near-infrared light, the *in vitro* proliferation of CT-26 cells was significantly inhibited due to increased apoptosis and cell cycle arrest. Moreover, the experimental results confirmed that the proliferation inhibition effect was dose-dependent. This novel drug delivery system, together with heat treatment, provides a feasible method for killing PD-L1-overexpressing cells. However, DOX is hydrophobic, and to improve its stability and selectivity, DOX-conjugated GNs are generally constructed by covalent attachment of many polyethylene glycol (mPEG) molecules with a thiol group at one end and a drug molecule at the other end 59, 60. When the drug molecules are attached to the GNs via PEG linkers, they are exposed on the surface of the conjugates and tend to be absorbed by various proteins, resulting in inefficient therapy. Ding et al. proposed a feasible method to change the relative position of PEG and drug to solve these issues associated with drug-conjugated GNs (4 nm) 61. They designed a novel lipoic acid (LA)-modified PEG derivative of DOX instead of using PEG as a linker. The carbonyl and amino groups of DOX were modified with LA and mPEG, respectively. This method improved the solubility, stability, and dispersion of the gold conjugate. Furthermore, following endocytosis of the nanoparticles into cells, the drug was released in two steps. DOX-mPEG was first released from the GNs in acidic lysosomes and then free DOX was generated in the cytoplasm by the catalytic activity of esterase (Figure 3). Thus, the cytoplasm was a reservoir for sustained drug release in tumor cells, and the gold conjugates showed an excellent antitumor effect compared to doxorubicin hydrochloride (DOX•HCl). This work offers a new perspective for improving the performance of nanocarriers by designing and modulating the basic structures of the gold conjugates.

Many other types of drug molecules have been reported to show improved therapeutic effects when coupled with ultrasmall GNs. Liang et al. synthesized ultrasmall GNs (2 nm) and successfully conjugated a therapeutic peptide (p12) and a targeting peptide (CRGDK) on the surface to study the bioeffects on breast cancer cells 11. The functionalized GNs selectively bound to Nrp-1 receptors, which mediated the internalization of the nanoparticles, thus improving the delivery of p12 inside the targeted cells. The functionalized GNs were highly effective against cancer cells, as the p12 therapeutic peptide was able to increase the expression of p53. Such drug delivery systems containing both drugs and targeting agents constitute hybrid platforms for cancer treatment. Similarly, Saini et al. studied the antitumor potential of nisin-, doxorubicin- and nisin-DOX-conjugated GNs (8-12 nm) on DMBA-induced (7,12-dimethylbenz(a)anthracene; DMBA) murine skin cancer 62. The results revealed that the conjugated GNs reduced the size of the skin cancer lesions. This effect was likely achieved by ROS-mediated apoptosis and immunomodulation either alone or synergistically. Such a combination of multiple chemotherapeutics reduced the chances of cancer cells developing drug resistance. Ehrlich et al. presented a means to enhance the sensitivity of drug-resistant tumor cells to chemotherapeutic drugs through delivery of GNs (2 nm) loaded with drugs and DNA 63. The research demonstrated that GNs carrying the Hsp90 (heat shock protein 90) inhibitor 17-AAG(17-N-allylamino-17-demethoxygeldanamycin) and DNA encoding Cullin-5 (Cul5) increased the sensitivity of Cul5-deficient AU565 cells to 17-AAG. This work provided evidence that drug-resistant tumor cells can be sensitized by delivering DNA with a drug. Coulter et al. assessed the ability of ultrasmall gold core nanoparticles (2 nm) conjugated with the potent maytansine analog DM1 (MTC-100038) to treat murine hepatocellular carcinoma 64. Mice treated with DM1 at a dose of 150 μg / kg showed weight loss, while mice treated with a MTC-100038 dose of 450 μg / kg still did not show significant weight loss or toxic side effects. Compared with free drug, the GN platform improved systemic tolerability and facilitated drug delivery to hepatocellular tumors following intravenous administration. Arunakaran et al. evaluated the effects of GNs (3 nm) conjugated with quercetin in MCF-7 and MDA-MB-231 breast cancer cell lines 65. The results indicated that quercetin-conjugated GNs were more potent than free quercetin and could be used for targeted delivery of drugs with enhanced therapeutic efficacy in combating breast cancer. The nanoparticles inhibited the phosphorylation of EGFR and the activity of downstream molecules in the PI3K/Akt pathway in breast cancer cells. These gold-based drug delivery systems provide evidence for the potential application of ultrasmall GNs in cancer chemotherapy.

### Radiotherapy

Radiotherapy is one of the common methods used to treat cancer. It relies on the use of high-energy radiation to kill tumor cells. Radiosensitizers can effectively increase the radiation dose at the cellular level and can therefore enhance the effect of radiotherapy. GNs have the potential to serve as an excellent radiosensitizer in radiotherapy. This is because GNs accelerate the breakage of DNA strands when exposed to gamma or X-rays 19, 20, 66. In order for GNs to be used effectively in radiation therapy, it is important to determine what size has the best therapeutic effect. Smilowitz et al. previously injected 1.9 nm-diameter gold particles into mice bearing subcutaneous EMT-6 mammary carcinomas for X-ray therapy 55. The results showed that ultrasmall GNs could achieve the high metal content in tumors necessary for radiotherapy. On this basis, Liang et al. carried out a detailed study on the effect of radiotherapy on GNs with different sizes. Their group performed *in vitro* and *in vivo* radiosensitization studies on 4.8, 12.1, 27.3, and 46.6 nm PEG-coated GNs 20. Compared with 4.8 and 46.6 nm particles, 12.1 and 27.3 nm GNs were more widely dispersed in cells and had better therapeutic effects, which caused the tumor to disappear almost completely. These experimental results can help in the selection of a more suitable size of GN for enhancement of radiotherapy.

While GNs have obvious advantages as radiosensitizers, there are some issues that hamper their use in radiotherapy. The common issue is how to achieve selective targeting and rapid clearance of GNs to reduce the radiation dose and decrease the exposure of healthy tissue 67. An ideal radiosensitizer will show enhanced tumor retention and play a major role in enhancing tumor radiotherapy 68. Liang et al. reported that GSH-coated ultrasmall gold nanoclusters (2 nm) act as a metabolizable radiosensitizer for highly effective cancer radiotherapy 69. Because of their ultrasmall hydrodynamic diameter and biocompatible surface, these particles preferentially accumulated in the tumor via the improved EPR effect, which lead to marked improvement of radiotherapy efficiency. After the treatment, the ultrasmall gold nanoclusters could be excreted from the body by the kidney, minimizing any potential side effects due to the accumulation of gold nanoclusters in the body. This work presents a new and promising type of radiosensitizer for cancer radiotherapy with excellent characteristics such as improved tumor accumulation, enhanced radiation effects, and efficient renal clearance. Untargeted radiosensitizers rely on the EPR effect for tumor accumulation, but significant levels of off-target accumulation still limit this approach for radiotherapy 70. Basilion et al. synthesized Au_25_ nanoclusters (1.5 nm) that specifically target a prostate-specific membrane antigen ligand. These clusters provide a high-affinity radiosensitizer for the targeted cancer tissues in mice and therefore significantly enhance the effects of X-ray irradiation 67. GNs of 1.5 nm in diameter were selected because they could be cleared by the kidneys within hours, which helped to reduce their accumulation in other organs and their potential gold-induced organ toxicity. This design provides a valuable idea for even further optimization of GNs to improve the outcome of radiation therapy.

**Figure 4:**
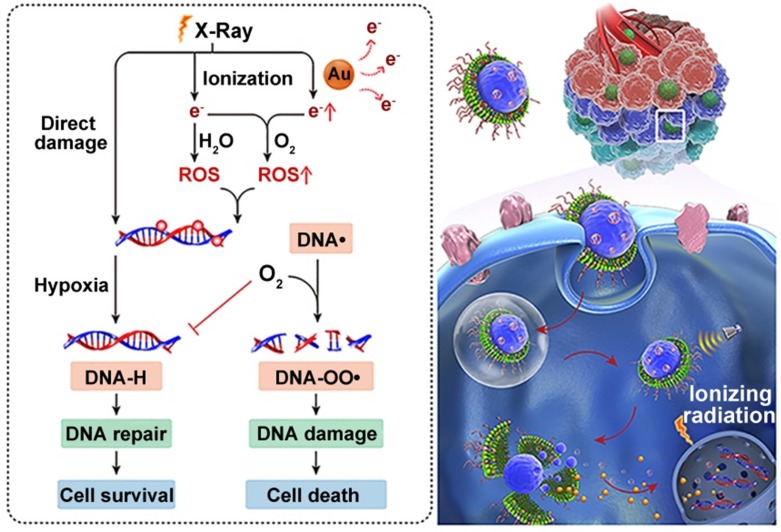
Ultrasmall GNs in cancer radiotherapy. Schematic representation of the mechanism of action of nanodroplets containing ultrasmall GNs for cancer radiotherapy. The right part shows the nanodroplets efficiently accumulating at the tumor site and further triggering a rapid release of oxygen and ultrasmall GNs upon ultrasound treatment. The left part shows how the ultrasmall GNs enhance DNA damage induced by radiotherapy, while the oxygen simultaneously relieves tumor hypoxia and fixes the DNA radical intermediates, consequently preventing DNA repair and eventually causing cancer cell death. Adapted with permission from 73, copyright 2018 American Chemical Society.

The treatment principle of radiotherapy is to damage intracellular DNA through the direct or indirect effects of external X-ray or γ-ray beams 71. Therefore, the effectiveness of radiotherapy is limited by insufficient DNA damage and rapid DNA repair during and after treatment 72. This issue has been a longstanding focus in translational radiotherapy research. To address this, Wang et al. created a hierarchical multiplexing nanodroplet for enhanced cancer radiotherapy through a DNA-dual-targeting approach 73. As shown in figure 4, the DNA-dual-targeting nanodroplet platform (NDr) can absorb the radiation energy and cause DNA lesions via the ultrasmall GNs (3.6 nm). The extent of DNA damage was confirmed by enhanced formation of γ-H2AX foci and the inhibition of tumor growth in a mouse model. Additionally, *in vitro* and *in vivo* studies confirmed that the effect of radiation therapy is dependent on the doses of ultrasmall GNs. Tumor growth was moderately inhibited with 5 mg / kg ultrasmall GNs. Importantly, O_2_ could be rapidly released from the liquid perfluorooctyl bromide core after ultrasound treatment. This may relieve the tumor hypoxia and prevent DNA repair by fixing the DNA radical intermediates, thus leading to cancer cell death. In conclusion, this dual-DNA-targeting strategy achieved an optimal outcome of radiotherapy.

### Photodynamic therapy

Photodynamic therapy (PDT) is a clinically approved cancer therapy which uses non-toxic dyes and harmless visible light in combination with oxygen to produce high level of ROS to kill cells 74. The hydrophobic nature of most photosensitizers makes them insoluble under physiological conditions and hinders their systemic administration 75, 76. Furthermore, the nonspecific distribution of photosensitizers in the body may cause serious adverse effects when patients are exposed to sunlight. GNs can carry drugs, while simultaneously providing improved stability and specific uptake into tumors, and have therefore been widely used for the delivery of photosensitizers for photodynamic therapy of cancer. Furthermore, the combination of metal nanoparticles and photosensitizer can enhance ^1^O_2_ production compared to the free photosensitizer 77.

**Figure 5:**
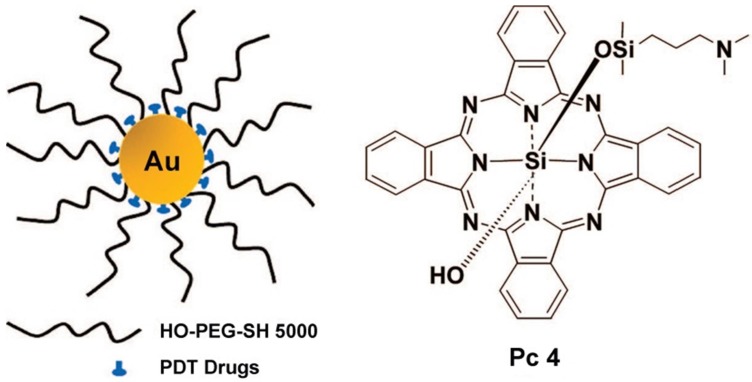
Ultrasmall GNs in cancer PDT. The left part shows a schematic representation of the GN-based PDT drug delivery system, which has greatly increased water solubility and reduced drug delivery time. PEGylated GNs are conjugated to the hydrophobic PDT drug Pc 4. The right part shows the structure of Pc 4. Adapted with permission from 78, copyright 2008 American Chemical Society.

Burda et al. used PEGylated gold nanoparticle (5 nm) to delivery hydrophobic drugs for PDT in a mouse model 78. This delivery mode greatly reduced the drug delivery time and improved the transport of the drug to the tumor (Figure 5). Burda et al. studied the drug delivery mechanism and pharmacokinetics of a system comprising non-covalent conjugates of a PDT cancer drug with GNs (5 nm) 79. This study indicated that the delivery of hydrophobic drugs into tumors in mice by passive accumulation of the GNs achieved rapid release and deep penetration into the tumor tissue. Burda et al. covalently attached the photoprecursor Pc 227 to GNs (5.5 nm) to generate the photodynamic therapy drug Pc 4 during 660 nm laser irradiation 80. This facilitated controlled drug release, which could maximize the accumulation of drugs in the targeted tissue. Pérez-García et al. synthesized a novel photosensitizer using dissymmetric porphyrin derivatives and incorporated it into GNs (3.5 nm) 81. The photosensitizer-loaded gold colloid nanoparticles improved the water solubility and activity of photosensitizer. These reports provided evidence that GNs can effectively overcome the hydrophobicity of photosensitizers and deliver them to tumor sites.

**Figure 6:**
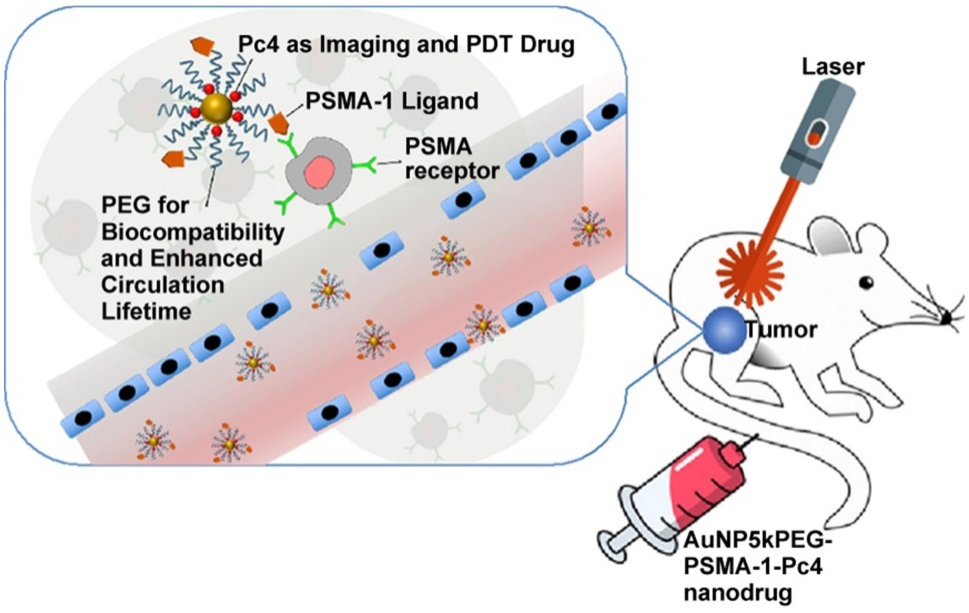
Ultrasmall GNs in PDT of prostate cancer in a mouse model. Schematic representation of ultrasmall GNs carrying the prostate-specific membrane antigen PSMA-1 for targeted delivery of the fluorescent PDT drug Pc 4 to prostate cancer cells. Pc 4 kills cancer cells when exposed to light. Adapted with permission from 84, copyright 2018 American Chemical Society.

Broome et al. achieved targeted therapy by constructing GNs (5 nm) conjugated with epidermal growth factor (EGF) peptide and the PDT drug Pc 4 82. The results suggested that intracellular uptake of GNs via EGF peptide increased the intracellular accumulation and therapeutic efficacy of Pc 4. Russell et al. functionalized GNs (3~5 nm) with a mixed monolayer of zinc phthalocyanine and a lactose derivative 83. The results showed that lactose acted as the stabilizing agent for the GNs loaded with the hydrophobic phthalocyanine photosensitizer, and as the targeting agent for breast cancer cells. These phthalocyanine-loaded GNs generated singlet oxygen during irradiation and caused cell death. Burda et al. developed a gold nanoparticle (5.5 nm) platform carrying PSMA-1 (prostate-specific membrane antigen-1) and the fluorescent photodynamic therapy drug Pc 4 84. These nanoparticles effectively deliver Pc 4 to prostate cancer cells, so that the tumor can be visualized and then destroyed when irradiated with light (Figure 6). Such a design can provide guidance for surgical treatment and postoperative intervention.

Deng et al. developed liposomes loaded with GNs (3~5 nm) together with a photosensitizer RB and the antitumor drug DOX 85. The results indicated that GNs encapsulated inside liposomes can contribute to enhanced ^1^O_2_ generation, and light illumination triggered drug release from the liposomes. Pérez-García et al. synthesized water-soluble GNs (7~10 nm) functionalized with mixed ligands (a polyethyleneglycol-containing thiol and a new amphiphilic gemini-type pyridinium salt) and the anionic photosensitizer Na-ZnTCPP 86. The results showed that the combination of Na-ZnTCPP and GNs can enhance ^1^O_2_ production compared to the free Na-ZnTCPP. This may be because the gold core enhanced the activity of porphyrin. These results showed that GNs can provide the additional benefit of enhancing the effects of photodynamic therapy effects as well as acting as delivery agents.

### Gene therapy

**Figure 7:**
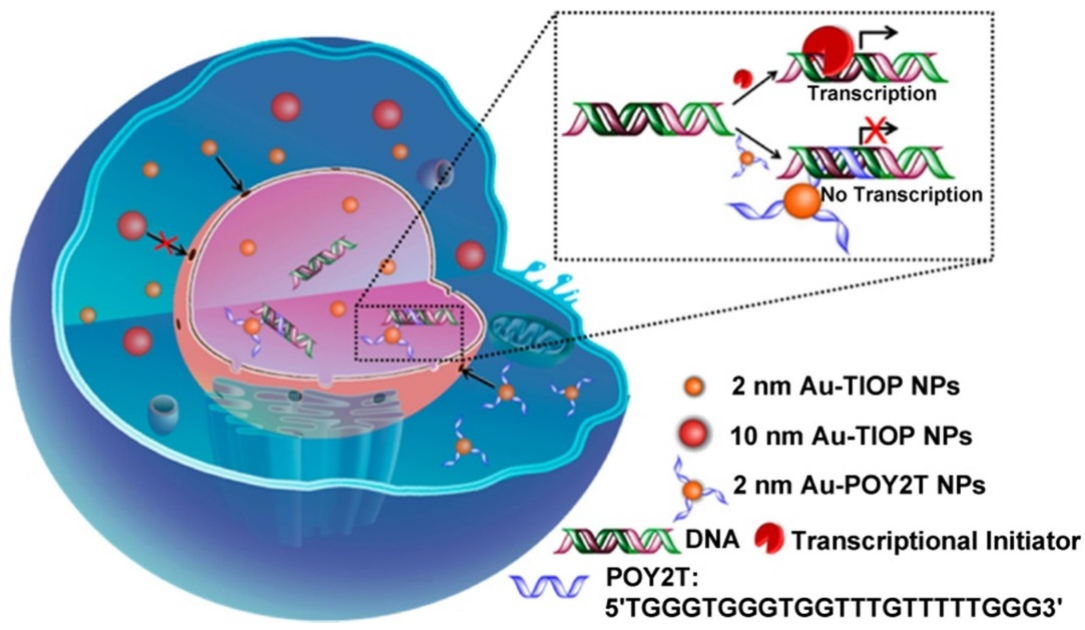
Ultrasmall GNs in cancer gene therapy. Schematic representation of the distribution and localization behavior of smaller (2 nm) and larger (10 nm) GNs in MCF-7 cancer cells. The ultrasmall 2 nm GNs were able to enter the nucleus, and were used as a carrier to deliver a triplex-forming oligonucleotide (TFO) to regulate gene expression. Adapted with permission from 88, copyright 2014 American Chemical Society.

Gene therapy, the strategy of using genetic materials to cure diseases, has great potential in cancer treatment 32. The design of an efficient gene delivery system is the key to gene therapy. The effect of size-dependent permeability of GNs on gene delivery has been investigated. Ultrasmall GNs can overcome the obstacles associated with gene delivery at the systemic and cellular levels, and they have been widely reported as gene delivery vectors 87. Rotello et al. synthesized GNs (2 nm) functionalized with triethylenetetramine-terminated dendrons as a vector for siRNA delivery 33. Liang et al. also explored this in detail. Their team first synthesized GNs with different sizes (2, 6, 10 and 16 nm) and researched their intracellular distribution in MCF-7 cells 88. The results showed that only the nanoparticles smaller than 10 nm (2 and 6 nm) were found in the nucleus (Figure 7). On this basis, they used ultrasmall 2 nm GNs to deliver triplex-forming oligonucleotides (TFOs). This delivery system was more effective at reducing cell viability than free TFO. This study also provided a new guide for selecting appropriately sized nanocarriers to deliver therapeutic substances to the nucleus to regulate gene expression.

**Figure 8:**
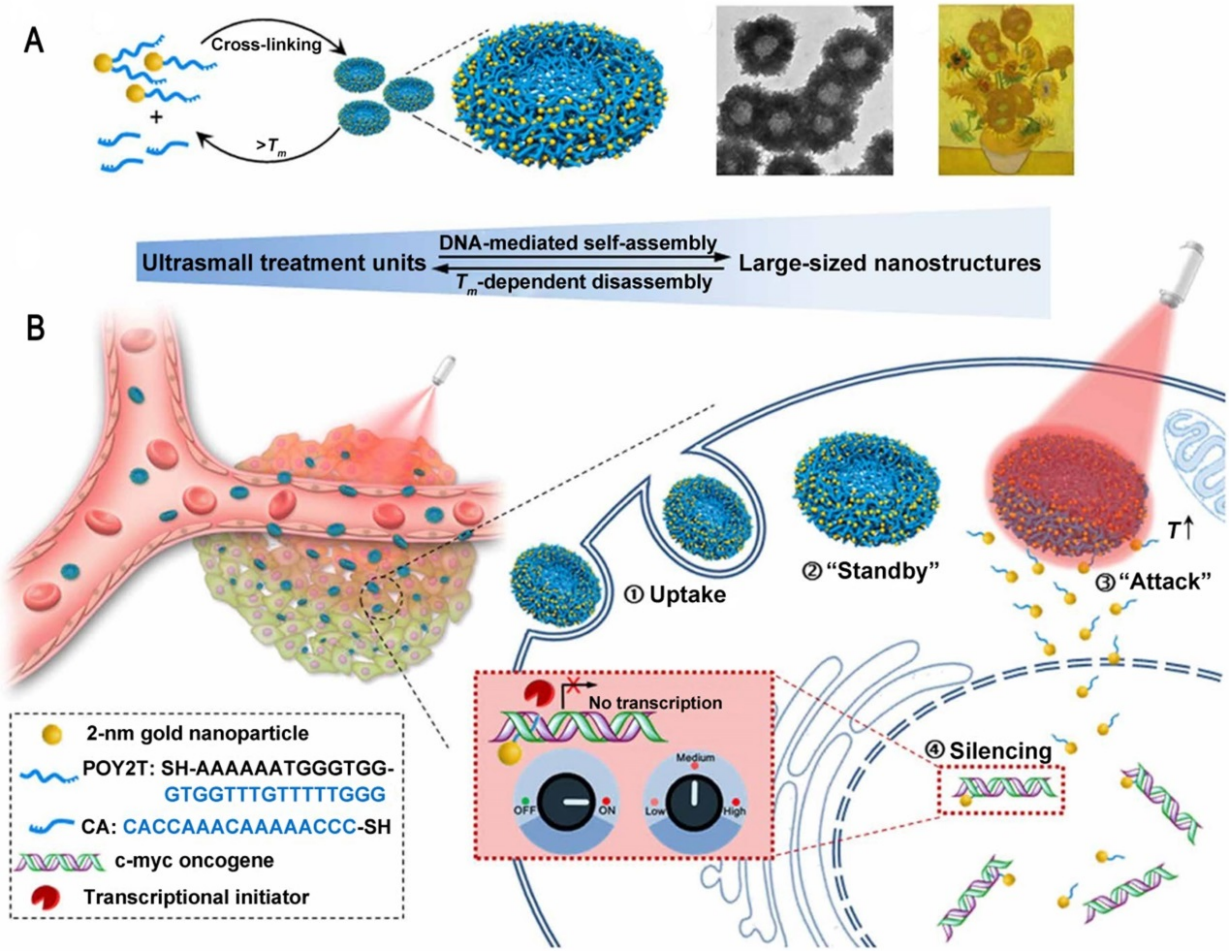
Ultrasmall GNs in cancer gene therapy. Schematic representation of the synthesis and mechanism of action of gold-DNA nanosunflowers for efficient gene silencing. The upper part (A) shows the reversible assembly of the large nanostructures from thiol-oligonucleotide-modified ultrasmall GNs. The lower part (B) shows that the large gold-DNA nanostructures dissociate upon NIR irradiation and release small units which enter the cell nucleus and silence the target oncogene (c-myc). Adapted with permission from 90, copyright 2019 The Authors.

There are still many challenges to overcome in terms of enhancing and controlling gene-based therapies. For example, therapeutic nucleic acids must be shielded from enzymatic and physical degradation. Rotello et al. designed a system in which trimethylammonium-modified GNs (2 nm) were combined with DNA by electrostatic interaction to protect the DNA from degradation 32. Mirkin et al. reported an oligonucleotide-modified GN (13 nm) to control intracellular protein expression through gene regulation 89. Importantly, the GNs improved the stability of the oligonucleotides and prevented their degradation by nucleases. In addition, the cellular uptake of the GN-oligonucleotide complexes was more than 99%, which meant that they were highly effective for gene regulation. Inspired by this work and other previous work, Liang et al. designed DNA-mediated self-assembled gold-DNA sunflower-like nanostructures with excellent properties including NIR absorption and photothermal conversion 90. Research results showed that the nanostructures have an excellent photothermal effect. As illustrated in figure 8, upon NIR irradiation, ultrasmall (2 nm) GNs were liberated from the large nanostructures and further delivered triplex-forming oligonucleotides into the nucleus to interfere with the gene transcription process. The released 2 nm GNs modified with a sequence to silence the c-myc oncogene exhibited improved nuclear permeability and thus an enhanced transfection efficiency. These transformable nanosunflowers provide an excellent model for designing efficient and tailorable nanocarriers for combination therapy.

### Other treatments

**Figure 9:**
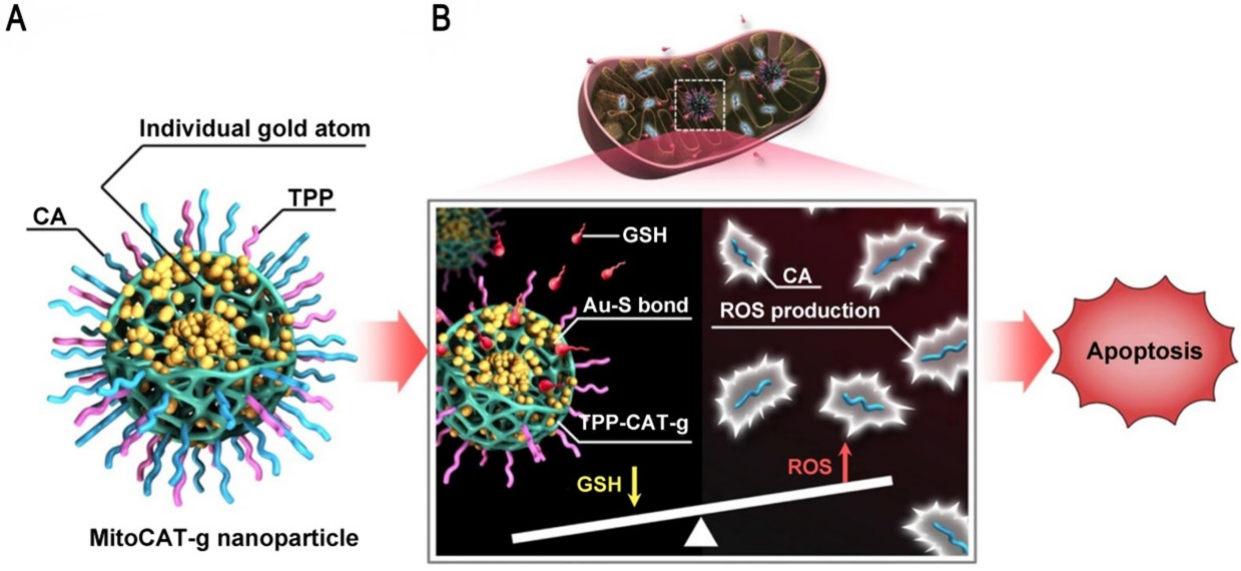
Ultrasmall GNs in other treatments: amplification of mitochondrial oxidative stress. Schematic representation of the design and mechanism of triphenylphosphine- and cinnamaldehyde-modified carbon dots containing gold atoms. After the nanoparticles are taken up by endocytosis, acid-responsive cinnamaldehyde is released and then generates ROS in mitochondria. Following cinnamaldehyde release, gold atoms consume GSH by forming Au-S bonds. The changes of ROS and GSH in cells eventually lead to apoptotic cell death. Adapted with permission from 93, copyright 2019 The Author(s).

Mitochondria are a potential target for cancer therapy, because mitochondrial damage leads to altered redox homeostasis, ROS production, and apoptotic cell death. However, intracellular GSH can rapidly neutralize the ROS produced in mitochondria 91, 92. GNs can be used to consume GSH in cells and the atomically dispersed gold atoms provide the highest atom utilization. Liang et al. reported a mitochondrial oxidative stress amplifier that used carbon dots as support materials for highly stable and well-dispersed gold atoms, which were further surface-modified with triphenylphosphine and cinnamaldehyde 93. This design used gold atoms to consume GSH, thus amplifying cinnamaldehyde-induced ROS damage and leading to apoptosis (Figure 9). This nanoparticle was a promising anticancer agent because it realized the consumption of GSH and the elevation of ROS in mitochondria at the same time.

A nanozyme is a nanomaterial with enzyme-like characteristics. The variable size and higher stability of nanozymes means that they may be more beneficial against solid tumors than natural enzymes, which are restricted by the chemical composition and temperature of the local environment 94, 95. Rossi et al. discovered that GNs are able to catalyze the oxidation of glucose to H_2_O_2_ and gluconic acid in the presence of dissolved oxygen. This indicated that GNs can be used as a substitute for glucose oxidase (GOx) because their activities are similar 96. This work laid the foundation for the use of GNs as nanozymes to treat tumors. Shi et al. reported a biomimetic dual inorganic nanozyme which triggers catalytic cascade reactions in the tumor microenvironment for nanocatalytic tumor-specific therapy 97. Ultrasmall GNs (1.5 nm) catalyzed the oxidation of glucose to produce H_2_O_2_, and the H_2_O_2_ was further converted into highly toxic hydroxyl radicals through a Fe_3_O_4_-based Fenton reaction which eventually caused cell death. Wu et al. selected a porphyrin metal-organic framework (PCN) with photodynamic therapy and fluorescent imaging abilities. They sandwiched catalase-mimicking Pt nanoparticles between PCN and then embedded ultrasmall GOx-mimicking GNs (2 nm) within the outer shell. The system was coordinated with FA to improve tumor accumulation 98. This nanoreactor achieved a markedly strengthened antitumor effect in a mouse model by enhancing photodynamic therapy and accelerating glucose depletion. In particular, the produced H_2_O_2_ acted as a substrate for the Pt nanoparticles. The studies described here highlight the unique potential of GNs as components of nanozymes for cancer treatment. They also provide ideas for designing catalytic cascade models for antitumor therapy.

## Conclusions and Perspectives

We have reviewed the advantages and biomedical applications of ultrasmall GNs with a spotlight on cancer treatment. Size is one of the key physical parameters of GNs, which directly affects their properties such as toxicity, biocompatibility, etc. Many reports have explored the size effect of GNs, and the results indicated that ultrasmall GNs have more effective renal clearance, higher tumor tissue permeability, better cell uptake and more efficient entry into nuclei. A better understanding of the properties of ultrasmall GNs will help to design ultrasmall gold-based nanoplatforms to further extend their biomedical applications. Surface modifications and functionalization have played key roles in the development of ultrasmall GNs. Currently, ultrasmall GNs with excellent optical and electrical properties have been used as contrast agents in magnetic resonance imaging, photoacoustic imaging, fluorescence imaging, and X-ray scatter imaging. Importantly, ultrasmall GNs can specifically target tumor tissues and deliver agents for photodynamic therapy, chemotherapy, gene therapy and so on, to enhance the efficiency of cancer cell killing and minimize the impact on non-tumor tissues.

Although the current results of research on ultrasmall GNs in biomedical applications are indeed encouraging, there are still issues that need more attention. First, based on the physical properties of GNs, more research is needed on integration of diagnosis and treatment. Secondly, as a universal challenge in cancer treatment, the differences between patients complicate the optimization of GNs for cancer therapy, and more efforts are needed to address this obstacle. However, the ease of functionalization of ultrasmall GNs offers more opportunities for personalized medicine. Given the success of ultrasmall GNs for biological imaging and cancer treatment, the development of future strategies to overcome obstacles and realize gold-dependent cancer cell killing is particularly promising. Taking all the evidence together, we believe that ultrasmall GNs offer unique opportunities to translate the insights of basic research into clinical applications.
